# Guillain-Barré syndrome related to Zika virus infection: A systematic review and meta-analysis of the clinical and electrophysiological phenotype

**DOI:** 10.1371/journal.pntd.0008264

**Published:** 2020-04-27

**Authors:** Sonja E. Leonhard, Cristiane C. Bresani-Salvi, Joanna D. Lyra Batista, Sergio Cunha, Bart C. Jacobs, Maria Lucia Brito Ferreira, Maria de Fatima P. Militão de Albuquerque

**Affiliations:** 1 Department of Neurology, Erasmus University Medical Center, Rotterdam, The Netherlands; 2 Laboratory of Virology and Experimental Therapy, Oswaldo Cruz Foundation, Ministry of Health, Recife, Brazil; 3 Medical Sciences College, Federal University of Fronteira Sul, Chapecó, Brazil; 4 Department of Preventive Medicine, Federal University of Pernambuco, Recife, Brazil; 5 Department of Immunology, Erasmus University Medical Center, Rotterdam, The Netherlands; 6 Department of Neurology, Hospital da Restauração, Recife, Brazil; 7 NESC Department, Oswaldo Cruz Foundation, Ministry of Health, Recife, Brazil; Australian Red Cross Lifelood, AUSTRALIA

## Abstract

**Background:**

The Zika virus (ZIKV) has been associated with Guillain-Barré syndrome (GBS) in epidemiological studies. Whether ZIKV-associated GBS is related to a specific clinical or electrophysiological phenotype has not been established. To this end, we performed a systematic review and meta-analysis of all published studies on ZIKV-related GBS.

**Methods:**

We searched Pubmed, EMBASE and LILACS, and included all papers, reports or bulletins with full text in English, Spanish or Portuguese, reporting original data of patients with GBS and a suspected, probable or confirmed recent ZIKV infection. Data were extracted according to a predefined protocol, and pooled proportions were calculated.

**Results:**

Thirty-five studies were included (13 single case reports and 22 case series, case-control or cohort studies), reporting on a total of 601 GBS patients with a suspected, probable or confirmed ZIKV infection. Data from 21 studies and 587 cases were available to be summarized. ZIKV infection was confirmed in 21%, probable in 22% and suspected in 57% of cases. ZIKV PCR was positive in 30% (95%CI 15–47) of tested patients. The most common clinical features were: limb weakness 97% (95%CI 93–99), diminished/absent reflexes 96% (95%CI 88–100), sensory symptoms 82% (95%CI 76–88), and facial palsy 51% (95%CI 44–58). Median time between infectious and neurological symptoms was 5–12 days. Most cases had a demyelinating electrophysiological subtype and half of cases were admitted to the Intensive Care Unit (ICU). Heterogeneity between studies was moderate to substantial for most variables.

**Conclusions:**

The clinical phenotype of GBS associated with ZIKV infection reported in literature is generally a sensorimotor demyelinating GBS with frequent facial palsy and a severe disease course often necessitating ICU admittance. Time between infectious and neurological symptoms and negative PCR in most cases suggests a post-infectious disease mechanism. Heterogeneity between studies was considerable and results may be subject to reporting bias. This study was registered on the international Prospective Register of Systematic Reviews (CRD42018081959).

## Author summary

Guillain-Barré syndrome (GBS) is a rare but severe neurological disease, characterized by an acute onset flaccid paralysis. GBS is thought to be caused by an exaggerated immune response to common infections that damages the peripheral nerves. The Zika virus (ZIKV) is the most recent pathogen to be connected to GBS, when large outbreaks of ZIKV infection in French Polynesia and Latin America were followed by an increased incidence of GBS patients. To better understand the clinical features and outcome of ZIKV-related GBS, we have performed a systematic review and meta-analysis of all published studies on GBS related to ZIKV. We identified 35 studies, reporting on a total of 601 patients with GBS and a suspected, probable or confirmed Zika virus infection, and were able to summarize data of 587 patients from 21 studies in a pooled analysis. Our study shows that published cases with ZIKV-related GBS generally have both sensory and motor symptoms, facial palsy, demyelination on electrophysiological examination, and a severe disease course that often necessitates ICU admittance. The relatively long time between infectious and neurologic symptoms and the lack of detection of viral particles in bodily fluids in most patients suggest a post-infectious rather than an infectious pathogenesis. However, these results should be interpreted taking into account the heterogeneity between studies, which was considerable for many variables, and a possible reporting bias of more severe cases. Outbreaks of ZIKV and GBS may appear in the future and our study can help clinicians in diagnosing and managing GBS patients in ZIKV endemic areas, and increases our understanding of the neuropathology of ZIKV.

## Introduction

Guillain-Barré syndrome (GBS) is the most common cause of acute flaccid paralysis worldwide, with an incidence rate of approximately 1 per 100,000 person-years.[1] GBS is an acute immune-mediated polyradiculoneuropathy, and is presumed to be triggered by preceding infections with specific pathogens, such as *Campylobacter jejuni*, cytomegalovirus (CMV), and Epstein-Barr virus (EBV).[2] Recently, the incidence of GBS increased during Zika virus (ZIKV) epidemics in French Polynesia (2013) and Latin America (2015–2016) and an association between GBS and ZIKV was established through epidemiological studies.[3, 4]

The classic form of GBS is characterized by a rapidly progressive and symmetrical weakness of the limbs, with sensory symptoms and reduced or absent tendon reflexes.[4] Cranial nerve involvement is frequent, with facial and bulbar muscles most often affected.[5] Electrophysiological studies help to confirm the diagnosis of GBS, and can indicate different subtypes, including acute inflammatory demyelinating polyradiculoneuropathy (AIDP), acute motor axonal neuropathy (AMAN), and acute motor and sensory axonal neuropathy (AMSAN).[4] The majority of patients will lose the ability to walk during the acute phase of the disease and about 25% of patients need to be mechanically ventilated at the Intensive Care Unit (ICU).[6] Clinical presentation and severity of GBS can vary extensively between patients. This variability is thought to be, in part, caused by differences in the type of preceding infections. For instance, *C*. *jejuni* has been associated with a pure motor axonal form of GBS with a severe disease course, while CMV has been linked to a sensorimotor GBS with pronounced respiratory insufficiency.[6–8]

Since the ZIKV epidemics, numerous studies have been published on ZIKV-related GBS, but it has not been established if there is a specific clinical and electrophysiological phenotype of GBS after ZIKV, and whether this differs from GBS triggered by other pathogens.[3, 4] Therefore, we have performed a systematic review and meta-analysis of all published studies on ZIKV-related GBS, and give a comprehensive overview of demographic characteristics, clinical features, diagnostic investigations, and outcome of ZIKV-related GBS patients.

## Methods

This systematic literature review follows the Preferred Reporting Items for Systematic Reviews and Meta-Analyses (PRISMA) statement and was registered on the international Prospective Register of Systematic Reviews (PROSPERO) with number CRD42018081959.[9]

### Information sources and search strategy

**Fig 1:**
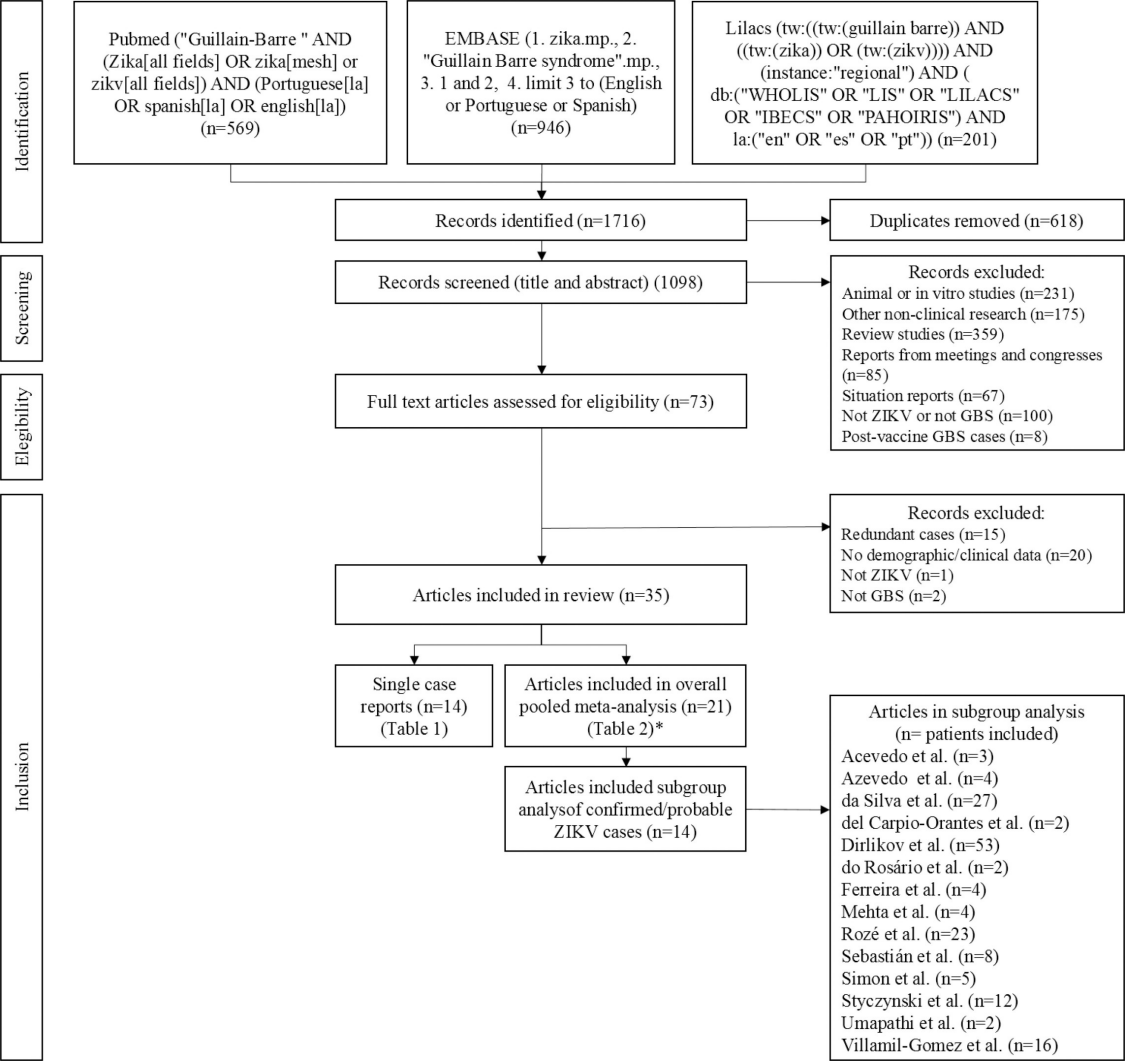
PRISMA Flowchart of search and selection of studies on GBS associated with recent ZIKV infection. *excluding Geurtsvankessel et al. (only one GBS case associated with a recent ZIKV infection).

First, by selecting key words from relevant articles, search strategies were constructed for the Pubmed, EMBASE and LILACS databases (**Fig 1**), which were searched on 24 November 2017 and on 24 January 2019. Second, the titles and abstracts were screened by two researchers (JDLB and SC) to identify the key words (‘Guillain-Barre Syndrome', ‘viruses’, ‘virus’, ‘Zika virus’ and ‘Zika’), and to exclude *in vitro* or *in animal* studies and reports from meetings or congresses. The selected papers were read in full by two independent reviewers (CCBS, MFPMA) and a third reviewer (SEL) was consulted in case of disagreement.

We included all papers, reports or bulletins with available full text in English, Spanish or Portuguese, without restriction in year of publication, reporting original data of patients with GBS and a suspected, probable or confirmed recent ZIKV infection, of any age, gender and in any setting. Predefined exclusion criteria were: GBS within 3 months after a vaccination or other proven triggering infection (e.g. *C*. *jejuni*), and studies with no information on age, residence, and at least one clinical variable of interest. When the study population of reported cases overlapped with cases published in other papers, the paper reporting the highest amount of cases was included. When only part of the cases in a study fulfilled our inclusion criteria, only these cases were included, but if separate data of these cases were not available after contacting the corresponding author, the article was excluded.

### Data extraction and management

Data were extracted independently by one of three reviewers (CCBS, MFPMA, SEL) according to a predefined protocol. The data extraction was then checked by one of the other two reviewers, and discrepancies were solved by discussion among all of them. Variables of interest comprised demographics, clinical characteristics (symptoms and signs of arbovirus infection and GBS), ancillary diagnostic investigations (electrophysiology and CSF), treatment, clinical course, and outcome of GBS. The corresponding authors were requested to share data on variables of interest that were not reported.

Cases were classified according to the reported diagnostic certainty levels for GBS and ZIKV infection. To classify the diagnosis GBS we employed the Brighton Collaboration Criteria (2011).[10] If the Brighton Criteria were not reported, these were defined based on available reported data, and if the clinical description did not correspond to the reported Brighton level, cases were reclassified after clarification was sought with the corresponding author. The diagnostic certainty of ZIKV infection was classified as confirmed, probable or suspected, according to the Centers for Disease Control and Prevention (CDC) criteria[11] (**Table 1**), based on the results of laboratory tests: case-by-case in case reports and series, and all cases combined in larger studies.

**Table 1:** Zika virus disease case definition.

*Suspected*	Acute onset of fever (measured or reported), OR maculopapular rash, OR arthralgia, OR conjunctivitis; OR Guillain-Barré syndrome (not explained by another etiology)*
*Probable*	Suspected ZIKV disease AND Epidemiologic linkage AND Laboratory evidence of recent ZIKV or flavivirus infection by: • Positive ZIKV IgM (serum/CSF) with: ° Positive neutralizing antibody titers against ZIKV and DENV (or other flaviviruses endemic to region of exposure) OR ° Negative DENV IgM and no neutralizing antibody testing performed.
*Confirmed*	Suspected ZIKV disease AND Laboratory evidence of recent ZIKV infection by: • Positive ZIKV culture, viral antigen or RNA (serum, CSF, tissue, or other specimen) OR • Positive ZIKV IgM (serum/CSF) with positive ZIKV and negative DENV (or other flaviviruses endemic to region of exposure) neutralizing antibody titers

Zika virus case definition according to the Centers for Disease Control (CDC).[11] ZIKV = Zika virus | DENV = Dengue virus | CSF = cerebrospinal fluid | RNA = Ribonucleic acid

*During a ZIKV epidemic

Clinical characteristics were retrieved as the number of patients in whom the variable was present in the numerator, and the total number of reported cases in the denominator: n/N (%). For arbovirus symptoms, we assumed symptoms were absent rather than missing if they were not cited in the manuscript, to account for the reporting bias, and therefore described as zero (n) out of the total number of reported cases (N). For the neurologic findings, variables not cited were considered missing data, because a risk of measurement bias was deemed higher than a risk of a reporting bias for these variables. If clinical characteristics were reported at multiple time points, data representing the full disease course were presented. Continuous variables (age, time between infectious and neurologic symptoms, duration of progression and plateau phase of GBS, duration hospital admission) were extracted as medians and or means, depending on how they were presented in the original article.

### Statistical analysis

First, we calculated the proportions per study of each variable of interest, and then the pooled proportions with data from all included studies reporting more than one GBS case. We were unable to summarize continuous variables, as in most studies these were reported as medians without availability of individual data or means. To address the possibility of an ascertainment bias of ZIKV infection among study populations, we then performed a subgroup sensitivity analysis, repeating the pooled analysis with grouped data of only probable or confirmed ZIKV cases (overall study populations comprising only probable/confirmed ZIKV cases, and subsamples of probable/confirmed cases from studies that also included suspected ZIKV cases, when available). We also performed sensitivity analyses by excluding papers that recruited only ICU patients, to account for selection bias in the pooled proportion of mechanical ventilation and ICU assistance.

The pooled proportions and the 95% confidence intervals (CI) were estimated using the random effects model and the Freedman Tukey double arcsine transformation, to account for proportions near 0 and 1. Heterogeneity between studies was calculated using the Chi-square test and I^2^ statistics, which was interpreted as follows: not important (I^2^ = 0–40%); moderate (I^2^ = 30–60%); substantial (I^2^ = 50–90%); considerable (I^2^ = 75–100%).[12] The meta-analysis was done using the *metaprop* command in STATA 15.1.[13]

## Results

### Study selection

We identified 1716 articles in the databases researched, of which 35 studies were included in our systematic review. The 35 selected studies reported on a total of 601 GBS cases with a suspected, probable or confirmed ZIKV infection with data of at least one variable of interest, and consisted of 13 single case reports and one cohort in which only one case fulfilled our inclusion criteria (n = 14, **Table 2**), and 14 case series and seven case-control studies (n = 587, **Table 3**). For the pooled analysis of the studies, we were only able to use the studies that reported on more than one case. (**Table 3**). For the subgroup meta-analysis of probable/confirmed ZIKV cases, data of 165 GBS cases with probable or confirmed ZIKV infection, from 14 studies, could be pooled (**Fig 1**).

**Table 2:** Single case reports of Guillain-Barré syndrome with recent ZIKV infection.

First author	Journal, year	City, country	Period	Clinical description	ZIKV diagnosis
Beattie[34]	Infect Dis Clin Pract 2018	Dominican Republic (DR)	2016	**64 y/o woman** returning from DR to USA. Paresthesias, sensory signs, tetraparesis, areflexia, difficulty walking, facial palsy. Preceding (10d) fever, rash, malaise, arthralgia, conjunctivitis, headache, cough, rhinorrhea. CSF: ACD. EMG: AIDP with axonal damage. Brighton level 1. Treatment: IVIg. ICU and MV. Discharge at 35d (tetraparesis).	ZIKV PCR+ (S,U) IgM: ZIKV- (S,CSF), DENV/CHIKV- (S) VNT ZIKV< DENV (S)
Brasil[35]	Lancet 2016	Rio de Janeiro, Brazil	Jun 2014	**24 y/o woman**. Paresthesias, tetraparesis, areflexia, difficulty walking. Concurrent fever, rash, headache, ocular pain, conjunctivitis, edema. Normal CSF and EMG. Brighton level 3. No treatment. Discharge at 13d (recovered).	PCR: ZIKV+ (S,CSF,U,Sa)PCR: DENV/CHIKV-(S, CSF)
Fabrizius[36]	Am J Trop Med Hyg 2016	Guyana	Mar 2016	**44 y/o man**. Paresthesias, sensory signs, ataxia, LL paresis, areflexia. Preceding (8d) fever, headache, rash, arthralgia, arthritis, conjunctivitis. CSF: ACD. EMG: sensorimotor peripheral neuropathy. Brighton 1. Treatment: IVIg. Discharge at 15d (walking with aid).	PCR: ZIKV/DENV/CHIKV- (S), ZIKV+ (U) IgM: ZIKV+ (S,CSF); DENV/CHIKV- (S) VNT ZIKV = DENV
Fontes[37]	Neuroradiol 2016	Rio de Janeiro, Brazil	2016	**51 y/o woman**. LL paresis, difficulty walking, facial palsy. Preceding (?d) rash, myalgia, arthralgia, conjunctivitis. CSF: ACD. EMG: AIDP. Treatment: IVIg. Clinical improvement. Discharge NR.	ZIKV+ (S,U–type tests NR)
Gonzalez-Escobar[38]	Rev Panam Salud Publica 2016	Tunapuna, Trinidad Tobago	Aug 2016	**29 y/o man**. Paresthesias, ataxia, LL paresis and progressing to tetraparesis. Preceding (7d) fever, rash, headache, malaise. Treatment: IVIg. Mild weakness at 10m.	PCR: ZIKV+(S), DENV/CHIKV-(S) IgM: ZIKV/DENV/CHIKV- (S)
Geurtsvankessel^a^[39]	Ann Clin Transl Neur, 2018	Dhaka, Bangladesh	Nov 2013-Dec 2015	**58 y/o woman.** Distal hypesthesia, tetraparesis, facial and bulbar palsy, autonomic symptoms (constipation). Treatment: IVIg. Independent walking at 3m.	IgM, IgG, VNT: ZIKV+ (S) PCR: ZIKV- (S)
Hamer[40]^b^	Ann Intern Med 2017	Suriname	May 2015-Feb 2016	**60 y/o woman** Returning from Surinam to the Netherlands. Tetraparesis, bulbar and bilateral facial palsy, areflexia, sensory signs. Preceding (?d) fever, myalgia, diarrhea, vomiting. CSF: ACD. EMG: AIDP. Brighton 1. Hospitalized 15d.	PCR: ZIKV+(U, S) IgM: ZIKV+(CSF).
Kassavetis[41]	Neurology 2016	Haiti	Jan 2016	**35 y/o man**. Paresthesias, sensory signs, bulbar and bilateral facial palsy, ophthalmoplegia, ataxia, areflexia. Preceding (1d) fever, headache, ocular pain, nasal congestion. CSF: ACD. Brighton 2 (MFS-GBS overlap). Treatment: IVIg. Discharge at 5d, walking with aid at 3w.	IgM&VNT: ZIKV+(S,CSF)
Miller[42]	J Neurol Sci 2017	Dominican Republic	May 2016	**55 y/o woman**. Paresthesias, LL paresis progressing to tetraparesis, bulbar and sensory signs, ataxia, areflexia. Concurrent asthenia, malaise, myalgia. CSF: ACD. EMG: AIDP. Brighton 1. Treatment: IVIg. Discharge at 22d (walking with aid).	PCR: ZIKV-(S,CSF,U) IgM: ZIKV+ (S,CSF), DENV+(S),CHIKV-(S) VNT ZIKV< DENV (S)
Rabelo[43]	Front Microbiol 2018	Rio de Janeiro, Brazil	Jun 2016	**28 y/o pregnant woman** (stillbirth). LL paresis progressing to tetraparesis, unable to walk, areflexia, paresthesias, sensory, autonomic, and respiratory signs. Preceding (20d) rash, vomiting. CSF: normal. EMG: AMSAN. Treatment: IVIg. Discharge at 28d, walking with aid at 40d.	ZIKV confirmed in placental and fetal tissues IgM: ZIKV/DENV/CHIKV- (S)
Raboni[44]	Transpl Infect Dis 2017	Maranhão, Brazil	Jun 2015	**9 y/o girl**. LL paresthesia, paresis, unable to walk, progressing to respiratory dysfunction. Preceding (90d) hematopoietic stem cell transplant. CSF: raised cell count and protein level. EMG: AIDP. Treatment: IVIg and PE. ICU, MV. Hospitalized (?d). Recovered at 4m.	PCR: ZIKV/DENV-(S), DENV NS1- IgM: ZIKV/DENV+(S) VNT ZIKV< DENV
Reyna-Villasmil[45]	Med Clin 2016	Zulia, Venezuela	2016	**28 y/o pregnant woman** (normal birth). Tetraparesis, bulbar palsy, areflexia, progressing to respiratory dysfunction. Preceding (10d) fever, rash, myalgia, conjunctivitis. CSF: ACD. EMG: AIDP. Brighton 1. Treatment: IVIg. ICU and MV. Discharge at 21d (recovered).	Serology for ZIKV+ (type tests NR)
Siu[46]	Neurology 2016	Tonga, Polynesia	2016	**47 y/o man**. Returning from Tonga to New Zealand. Paresthesias, progressive tetraparesis, areflexia, sensory and respiratory signs. Preceding (6d) edematous leg with pustular lesions. CSF: ACD. EMG: AIDP. Brighton 1. Treatment: IVIg and PE. ICU and MV. Discharge at 33d (bedbound).	PCR: ZIKV/DENV/CHIKV-(CSF)ZIKV+/DENV/CHIKV-(S), DENV NS1- IgM: ZIKV/DENV+(S)
Zambrano[47]	Am J Trop Med Hyg 2016	Guayaquil, Ecuador	Mar 2016	**57 y/o woman**. Paresthesia, facial palsy, tetraparesis, areflexia. Preceding (5d) headache, fever, lumbar back pain. CSF: ACD. Treatment: PE. ICU. Discharge at 10d.	PCR: ZIKV/CHIKV+/DENV- (S,CSF,U)

NR = Not Reported | y/o = year-old | USA = United States of America | LL = lower limbs | UL = upper limbs | ICU = Intensive Care Unit | MV = mechanical ventilation | CSF = cerebrospinal fluid | ACD = albuminocytological dissociation | EMG = electromyography/nerve conduction studies | IVIg = intravenous immunoglobulin | PE = plasma exchange | Brighton = Brighton Collaboration Criteria level | MFS = Miller Fisher Syndrome | ZIKV = Zika virus | CHIKV = chikungunya virus | DENV = dengue virus | PCR = polymerase chain reaction | VNT = virus neutralization test | DENV NS1 = NS1 antigen of DENV | S = serum | Sa = saliva | CSF = cerebrospinal fluid | U = urine.

^a^Not published as a case report but only one case fulfilling our criteria for suspected/probable/confirmed ZIKV in larger cohort of 418 cases.

^b^Returning travelers with suspected, probable or confirmed ZIKV infection reported to the GeoSentinel Surveillence Network. 93 cases reported, 2 GBS cases, one is already described in the Kassavetis’ paper, the other is described here.

**Table 3:** Demographic characteristics, case selection and ascertainment in studies reporting more than one Guillain-Barré syndrome case with a recent Zika virus infection.

First author	Journal, year	Provenance (city, country)	ZIKV outbreak	Study design	Incidence period	Study population	Ascertainment GBS^a^	Ascertainment ZIKV	N cases in analysis (mal:fem)	Median age (IQR) or [range]
Cao-Lormeau^**b**^[4]	Lancet 2016	Papeete, Tahiti,French- Polynesia	Oct 2013-Apr 2014	Prospective case-control	Oct 2013 - Mar 2014	All GBS inpatients in French Polynesia during ZIKV outbreak	Brighton 1–3 by neurologist or intensivist	PCR: ZIKV(S) VNT,IgM&IgG: ZIKV,DENV(S)	42 (11:31)	42 (36–56)
Simon[23]	J Neurovirol 2018	Noumea, New Caledonia, Melanesia	Jan-Dec 2014	Prospective case-control	Jan—Dec 2014	All GBS adult patients in New Caledonia during ZIKV outbreak	Brighton 1–2	PCR, IgM&IgG: ZIKV, DENV(S) VNT: ZIKV(S)	5^**c**^ (3:2)	52 (mean) [29–75]
Ferreira[16]	Am J Trop Med Hyg 2016	Recife, Brazil	Nov 2014–2015	Case series	15 Dec 2014–30 Jun 2015	First six adults with acute neurological illness and ZIKV PCR+, in reference neurology hospital	Criteria NR, data compatible with Brighton 1 and 4 (2:2)	PCR: ZIKV, DENV(S) IgM&IgG: ZIKV, DENV(S)	4 (1:3)	33.5 [25–48]
Nóbrega[29]	Epidemiol Serv Saude 2018	Recife, Brazil	Nov 2014–2015	Case series	23 Dec 2014–19 Jun 2015	All GBS inpatients in metropolitan region identified in the Hospital Information System, with arboviral symptoms (<60d) and/or laboratory positivity	Brighton 1–4 by medical records review	ZIKV PCR tested in 1 case (S) DENV IgM tested in1 case (S)	18 (9:9)	44 [14–62]
Styczynski[27]	PLoS Negl Trop Dis 2017	Salvador, Brazil	Jan 2015-May 2016	Retrospective case-control	1 Jan 2015–31 Aug 2015	All GBS cases (≥ 12y/o) reported to the Bahia Epidemiologic Surveillance Center	Brighton 1–3 by medical records review	IgM: ZIKV, DENV(S) VNT: ZIKV, DENV(S)	50^**d**^ (19:22)	44 [32–54]
do Rosário[17]	Am J Trop Med Hyg 2016	Salvador, Brazil	Jan 2015-May 2016	Case series	15 May - 30 Jul 2015	Adult patients admitted to ICU with ascending paresis, preceding exanthema, ZIKV IgM+	Wakerley Criteria, 2014	PCR: ZIKV, DENV, CHIKV(S) IgM&IgG: ZIKV, DENV, CHIKV (18 arboviruses panel in S) VNT:ZIKV, DENV, CHIKV, YFV(S)	2 (1:1)	46,5 [22 and 49]
Keesen[30]	Lancet 2017	João Pessoa, Brazil	2016	Case series	2016	GBS cases in Paraiba province admitted to neurology reference hospital during the ZIKV epidemic in 2016	NR	PCR: ZIKV IgM&IgG: DENV,CHIKV (type biosample NR)	12 (8:4)	35,5 [7–73]
da Silva[18]	JAMA Neurol 2017	Rio de Janeiro, Niteroi and São Gonçalo, Brazil	May 2015-Nov 2016	Cohort	5 Dec 2015- 10 May 2016	All adults with <60d onset of transverse myelitis, meningo-encephalitis or GBS admitted to neuromuscular expertise center	Brighton	ZIKV PCR if -: ZIKV IgM(S,CSF) ZIKV IgM if+: DENV IgM	28^**e**^ (9:19)	42 (22–67)
Azevedo[19]	Rev Soc Bras Med Trop 2018	Rio de Janeiro, Brazil	May 2015-Nov 2016	Case series	Jun 2015 - Dec 2016	All non-congenital neurologic disorders reported to Information System for Notifiable Diseases and Arboviral Neurologic Manifestation Report	PAHO criteria	PCR: ZIKV,CHIKV(S) IgM: DENV,CHIKV(S) IgG: CHIKV(S)	72 (NR)	45
Mehta[21]	PLoS Negl Trop Dis 2018	Rio de Janeiro, Brazil	May 2015- Nov 2016	Case series	1 Nov 2015–1 Jun 2016	Patients ≥12 y/o admitted to one of 11 participating hospitals, with acute neurologic disease, suspected and tested for ZIKV	Brighton by medical records review	PCR:ZIKV,DENV,CHIKV(S,CSF,U) IgM&IgG: ZIKV(S), DENV,CHIKV (S,CSF)	7^**f**^ (4:3)	41 [19–67]
Sebastián[22]	J Crit Care 2017	7 Latin American countries^**g**^	2015–2016	Case series	1 Dec 2015- 2 Apr 2016	Adults with a confirmed ZIKV infection in one of 24 ICUs of the Latin America Surveillance Network	Brighton by intensivist or neurologist (results NR)	PCR: ZIKV(S)	8 (2:6)	38 [18–67]
Salinas[24]	J Neurol Sci 2017	Barranquilla, Colombia	Oct 2015-Apr 2016	Retrospective case-control	1 Oct 2015- 2 Apr 2016	All GBS cases in Barranquilla reported to the national and the local surveillance system^**h**^	Brighton 1–3 by medical records review	IgM&VNT: ZIKV, DENV(S)	47 (25:22)	49 [10–83]
Parra[32]	NEJM 2016	Cucuta, Medellin, Cali, Barranquilla, Neiva, Colombia	Oct 2015-Apr 2016	Prospective case-control	Jan—Mar 2016	All patients with GBS at six university-based hospitals	Brighton by neurologist or internist	PCR: ZIKV(S,CSF,U), DENV(S,CSF) IgM&IgG: DENV(S,CSF)	68^**i**^ (30:38)	47 (35–57)
Villamil-Gomez[28]	Travel Med Infect Dis 2017	Sucre, Colombia	Oct 2015-Apr 2016	Case series	2016	Adults with confirmed ZIKV infection and GBS, admitted to ICU of two major clinical reference centers in Sincelejo-Sucre	NR	PCR: ZIKV; DENV NS1 IgM&IgG: DENV,CHIKV (samples NR)	16 (4:12)	53 (47–68)
Acevedo[20]	Front Microbiol 2017	Guayaquil, Ecuador	Jan-Oct 2016	Case series	1 Feb—31 Aug 2016	16 adult patients with neurological symptoms and PCR+ ZIKV, DENV or CHIKV in CSF, admitted to ER or ICU of largest hospital of Guayaquil	Criteria NR, data compatible with Brighton 1, 2, 4	PCR:ZIKV,DENV,CHIKV(CSF)	3 (1:2)	54 [18–62]
Langerak[31]	Front Neurol 2016	Paramaribo, Suriname	Oct 2015–2016	Cases series	Jan—Mar 2016	Consecutive adult patients diagnosed with GBS and preceding ZIKV infection	Criteria NR, data compatible with Brighton 1	PCR: ZIKV(S,CSF,U); IgM&IgG: ZIKV,DENV(S) VNT: ZIKV(S), DENV NS1(S)	3 (0:3)	50 [40–60]
Dirlikov-a[33]	MMWR 2016	Puerto Rico	Dec 2015-Dec 2016	Case series	1 Jan—31 Jul 2016	GBS cases admitted at 13 hospitals, identified by the GBS Passive Surveillance System-Puerto Rico Department of Health	Brighton by medical records review	PCR: ZIKV,DENV,CHIKV(S,CSF) IgM: ZIKV,DENV,CHIKV(S,CSF)	34 (20:14)	55 [21–88]
Dirlikov-b[25]	JAMA Neurology 2018	Puerto Rico	Dec 2015-Dec 2016	Case series	Jan—Dec 2016	All GBS cases admitted at all the 57 general hospitals of Puerto Rico and identified by the GBS Passive Surveillance System.	Brighton1-3 by medical records review	PCR: ZIKV,DENV,CHIKV (S,CSF,U,Sa) IgM: ZIKV,DENV,CHIKV(S,CSF)	107^**j**^ (47:60)	54 [4–88]
Rozé[26]	Clin Infect Dis 2017	Martinique, French Caribbean	Jan-Oct 2016	Case series	Jan—Oct 2016	All GBS inpatients at only specialized center in the country	Brighton 1–2 by neurologist	PCR:ZIKV,DENV,CHIKV (S,CSF,U) IgM&IgG: ZIKV,DENV,CHIKV(S) VNT ZIKV (if ZIKV PCR-&IgM-or ZIKV&DENV IgM+)	30^**k**^ (8:15)	61 (56–71)
del Carpio-Orantes[14]	Neurología 2018	Veracruz, Mexico	2016	Case series	2016–2017	All GBS cases documented by *Instituto Mexicano del Seguro Social* with GBS and tested for arboviruses	Brighton 1–3 by medical records review	PCR: ZIKV,DENV,CHIKV(S) IgM&IgG: ZIKV(S) IgM: DENV/CHIKV(S)	18	47 [19–70]
Umapathi[15]	J Peripher Nerv Syst 2018	Singapore, Singapore	Aug-Nov 2016	Prospective case-control	May—Dec 2016	All GBS cases from all public and private hospitals in Singapore before and during ZIKV outbreak	ICD10 G61.0 records in electronic databases	PCR: ZIKV,DENV(S,U) VNT, IgM&IgG: ZIKV,DENV(S)	12^**m**^ (7:5)	55,5 [25–81]
**Total**									**587**	

Age as median and IQR (interquartile range) or [range] unless indicated otherwise. mal = male | fem = female | NA = not applicable | Brighton = Brighton Collaboration Criteria levels[10] | EMG = electromyography/nerve conduction studies | y/o = years old | ICU = Intensive Care Unit | ZIKV = Zika virus | CHIKV = Chikungunya virus | DENV = Dengue virus | PCR = polymerase chain reaction | VNT = virus neutralization test | DENV NS1 = DENV NS1 antigen | NR = Not Reported | S = serum | CSF = cerebrospinal fluid | U = urine | Sa = saliva ER = emergency room | ICD10 = 10^th^ revision of the International Statistical Classification of Diseases and Related Health Problems).

^a^Patients not fulfilling the Brighton Criteria were included: da Silva (n = 3), Mehta (n = 1), Parra (n = 6).

^b^Additional data retrieved from previous publication by Watrin et al, 2016[48].

^c^Clinical data available for 5 cases with laboratory evidence of ZIKV infection (IgM & IgG positive).

^d^Age, infectious symptoms and laboratory data available for 41 cases included in case-control study, neurologic signs and symptoms available for all 50 reported cases.

^e^One post-vaccine case was excluded from data extraction, data on CSF examination were available for all 29 cases, age and clinical data were available for 27 ZIKV positive cases.

^f^A total of 13 GBS cases with suspected/probable/confirmed ZIKV were reported but data were available for only 7 cases with positive arbovirus tests.

^g^Colombia, Venezuela, Salvador, Guatemala, Puerto Rico, Ecuador, Perú and Chile.

^h^ Colombia National Surveillance System (Sivigila) and *Secretaria de Salud de Barranquilla*.

^i^Five cases from Barranquilla may overlap with cases reported by Salinas *et al*.

^jf^A total of 123 GBS cases with suspected/probable/confirmed ZIKV were reported but clinical and laboratory data were available for 107 cases tested for ZIKV.

^k^Laboratory data available for all cases and clinical data for 23 cases with laboratory evidence of ZIKV.

^l^Clinical data of 8 cases additionally retrieved from previous publication by del Carpio-Orantes *et al*, 2017.[49]

^m^A total of 14 cases were reported, data were extracted from 11 cases collected during the ZIKV outbreak plus one case with laboratory evidence of recent ZIKV infection before the outbreak.

### Study characteristics: case selection, case ascertainment and risk of bias

In **Table 2**, the single case reports are presented alphabetically with a brief clinical description per case. Eleven cases were from ZIKV epidemic or endemic regions and three were travelers returning from epidemic regions. Eight cases were positive for ZIKV PCR, four for IgM and plaque-reduction neutralization test (PRNT), and two were reported to be ZIKV positive with no further information provided. Six of eight cases of whom the Brighton classification was reported, fulfilled level 1. The most frequent clinical phenotype was a demyelinating sensorimotor GBS with facial and/or bulbar palsy.

In **Table 3**, the 21 studies reporting more than one patient are displayed according to the location and time-period of cases, in line with the global spread of the ZIKV epidemics on the Pacific islands (Oct 2013-Dec 2014) and Latin America (Dec 2014–2017). The first study was from French Polynesia in 2013–2014,[4] and the last was from Mexico in 2016–2017.[14] One study reported cases during and outside of a ZIKV outbreak period in Singapore[15]

Inclusion criteria, case selection and setting differed between studies. A diagnosis of GBS was the inclusion criterion in 14 studies, and seven studies also included other acute neurologic illnesses besides GBS.[16–22] Six studies included all GBS patients in their reference population,[4, 15, 23–26] one study included all GBS patients >12 years old,[27] and one study included all arbovirus-related neurologic manifestations.[19] All other studies included a convenience sample of patients seen at one or more health-care centres. Three studies only included patients admitted to the ICU,[17, 22, 28] and nine studies only included GBS patients with a clinical suspicion or laboratory evidence of a ZIKV infection.[14, 17, 20–22, 28–31] Seven studies were set in a specialized hospital (academic or reference centre),[4, 16, 18, 23, 26, 31, 32] and two multi-centre studies were set in both specialized and non-specialized hospitals.[21, 28] These differences are potential sources of selection bias within studies and heterogeneity across studies.

Sixteen studies reported the criteria that were applied for diagnostic certainty of GBS, and 13 used the Brighton Criteria. In four studies the Brighton Criteria were prospectively applied by a physician; in seven, retrospectively through records review; two studies gave no information on how the Brighton level was assessed; and three employed other criteria. The risk of ascertainment bias of GBS is likely to be low or very low, as the vast majority of all cases with this data available in this review fulfilled Brighton levels 1–3 (396/407, 97%).

Regarding the ascertainment of ZIKV infection, 13 studies tested their cases for both PCR and IgM,[4, 14–18, 21, 25, 26, 28, 30, 31, 33] five only for PCR,[19, 20, 22, 29, 32] and three only for IgM.[23, 24, 27] Based on the CDC ZIKV case definition, more than a half of all GBS cases with this data available had a suspected ZIKV infection (324/570, 57%), which gives a high risk for ascertainment bias within studies and heterogeneity across studies.

### Patient characteristics

#### Demographics

The median age of the study populations varied between 34 and 61 years, and only 11 pediatric patients were included in four studies.[19, 24, 27, 30] The majority of patients was male (62%) and the male:female ratio of all studies combined was 1.63. In multicenter studies or those including all GBS cases in the reference population, the male:female ratio was 1:1, with the exception of studies from French Polynesia[4] and Martinique[26], which had ratios of 3:1 and 2:1, respectively (**Table 3**).

#### Certainty levels of GBS diagnosis and ZIKV infection

**Fig 2:**
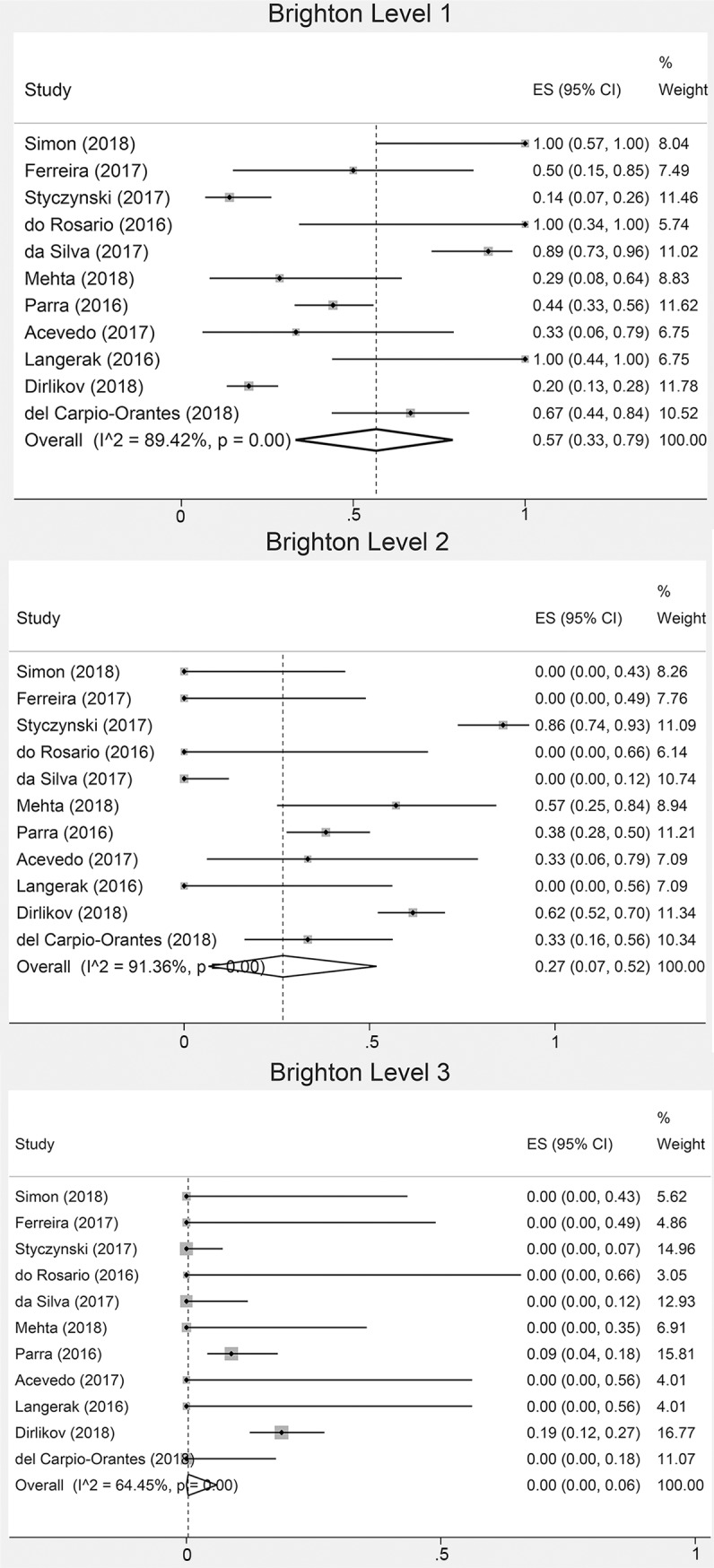
Overall pooled proportions (forest plots) of Brighton classification of GBS cases during ZIKV epidemics.

Separate proportions of each Brighton level (1–4) were available in ten studies[14, 16–18, 20, 27, 31–33] (295 cases): 110 cases fulfilling level 1; 146 level 2; 26 level 3 and 13 level 4. Miller Fisher Syndrome (MFS) was reported in only four studies: one study from Singapore (five cases),[15] and three studies from Latin America (six cases).[14, 18, 32] ZIKV infection was confirmed in 118 (21%), probable in 128 (22%) and suspected in 324 (57%) of all cases with reported separate proportions of each ZIKV certainty level. In the overall pooled estimates of study populations with available proportions of at least the Brighton level 1 and a suspected ZIKV infection, 57% of cases had Brighton level 1 and 44% had a suspected ZIKV infection (**Fig 2**, **S2 Fig**). We re-calculated these pooled frequencies after excluding two studies that only included cases with Brighton levels 1–2,[23, 26] finding 51% (95%CI: 28–74; I^2^ 89.2%) with Brighton 1 (105/290), and re-calculated pooled frequencies after excluding eight studies that only included cases with probable/confirmed ZIKV,[16, 17, 20–23, 28, 31] finding 65% (95%CI: 47–80; I^2^ 93.2%) with a suspected ZIKV infection (319/522).

#### Clinical characteristics

**Fig 3:**
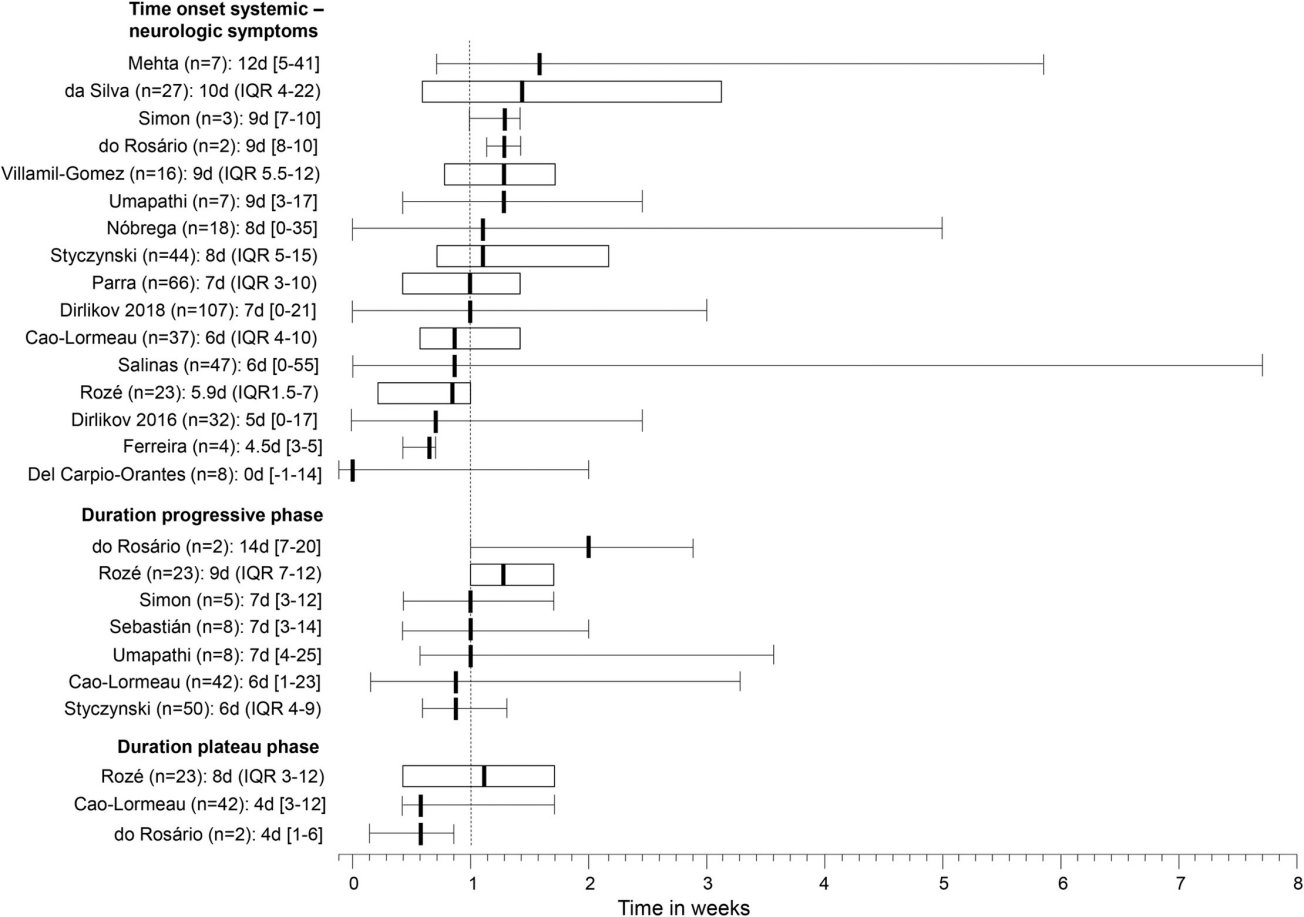
Per study medians and ranges of days of time between onset of infectious and neurologic symptoms, and the progressive and plateau phase of GBS cases. () = inter quartile range, [] = range.

All but one study reported the presence of clinical symptoms of infection.[19] Two or more symptoms were present in 91% of cases (378/444; 95%CI 84–96, I^2^ 61.2%). The most common symptoms were rash, fever and arthralgia, with similar pooled frequencies between overall estimates and the probable/confirmed subgroup (**Table 4**). The median time between the start of infectious symptoms and neurologic symptoms ranged from -1 to 12 days in the 16 studies reporting on this (**Fig 3**). For arbovirus symptoms the heterogeneity ranged from considerable (I^2^ = 75–100%), in the overall analysis, to substantial (I^2^ = 50–90%), in the probable/confirmed subgroup.

**Table 4:** Demographics and clinical characteristics of GBS cases associated with ZIKV reported in 21 case series.

	All cases (N 587)		Probable/confirmed ZIKV infection (N 165)	
**Demographics**				
Adults % (n/N)	98% (550/563)		100% (165/165)	
Female % (n/N)	38% (216/570)		41% (67/165)	
**Symptoms**				
**Infectious symptoms**	**n/N**	**Pooled proportion (95%CI; I**^**2**^**)**	**n/N**	**Pooled Proportion (95%CI; I**^**2**^**)**
Arboviral symptoms				
Rash	253/544	56% (43–69; 83%)	86/149	61% (37–82; 78%)
Fever	228/539	45% (33–57; 77%)	66/149	42% (21–64; 75%)
Arthralgia	150/539	35% (21–49; 86%)	50/149	31% (15–50; 64%)
Myalgia	126/550	25% (12–41; 89%)	40/149	29% (7–55; 83%)
Headache	106/550	22% (8–38; 91%)	32/149	25% (5–50; 83%)
Conjunctivitis	98/539	17% (8–28; 80%)	30/149	15% (7–24; 14%)
Ocular pain	24/550	1% (0–6; 74%)	3/149	0% (0–3; 41%)
Gastrointestinal^a^	59/550	8% (3–14; 66%)	15/149	6% (0–21; 67%)
Rhinorrhea	12/550	0% (0–1; 0%)	1/149	0% (0–0; 0%)
Cough or chest pain	28/550	2% (0–7; 71%)	9/149	2% (0–13; 61%)
**Neurologic symptoms**	**n/N**	**Pooled proportion (95%CI; I**^**2**^**)**	**n/N**	**Pooled proportion (95%CI; I**^**2**^**)**
Sensory symptoms	333/421	82% (76–88; 30%)	97/119	86% (73–96; 34%)
Dysphagia	133/351	30% (17–45; 90%)	49/112	34% (7–67; 85%)
Dysarthria	64/281	11% (1–25; 78%)	3/13	17% (0–60; 48%)
Diplopia	11/234	0% (0–4; 33%)	1/13	2% (0–25; 0%)
**Neurologic signs**	**n/N**	**Pooled proportion (95%CI; I**^**2**^**)**	**n/N**	**Pooled proportion (95%CI; I**^**2**^**)**
Facial palsy	246/486	51% (44–58; 36%)	75/139	56% (42–71; 38%)
Bulbar palsy	60/182	25% (10–42; 70%)	4/11	32% (0–76; 33%)
Ocular palsy	22/232	5% (0–12; 46%)	0/11	0% (0–19; 0%)
Any limb paresis	544/582	97% (93–99; 49%)	153/165	98% (93–100; 17%)
Tetraparesis	153/251	64% (51–77; 53%)	79/110	74% (61–87; 25%)
Paraparesis	69/251	24% (18–31; 0%)	21/110	15% (7–24; 0%)
Sensory deficits	155/317	49% (29–68; 86%)	59/104	59% (39–78; 48%)
Areflexia or hyporeflexia	400/435	96% (88–100; 79%)	131/142	97% (86–100, 56%)
Ataxia	76/317	17% (4–35; 87%)	34/91	29% (4–61; 74%)	
Respiratory dysfunction^b^	124/369	23% (13–35; 77%)	37/104	24% (10–41; 38%)
Dysautonomia	73/359	13% (5–24; 71%)	21/102	16% (8–26; 0%)
**GBS classification**	**n/N**	**Pooled proportion (95%CI; I**^**2**^**)**	**n/N**	**Pooled proportion (95%CI; I**^**2**^**)**
Brighton criteria				
Level 1–3	396/407	100% (97–100; 56%)	128/135	99% (93–100; 49%)
Level 4	13/407	0% (3–100; 62%)	7/135	1% (0–11; 54%)
Miller Fisher Syndrome	11/419	0% (0–2; 53%)	1/137	0% (0–0; 0%)
Other variants	3/419	0% (0–0; 0%)	0/137	0% (0–0; 0%)

Brighton level = Brighton Collaboration Criteria[10] levels.

^a^Nausea, vomiting or diarrhea.

^b^Reported as ‘trouble breathing’, ‘difficulty breathing’ or ‘respiratory dysfunction’

Among neurologic findings, paresis was reported in all studies, and almost all studies reported on sensory symptoms, tendon reflexes, and facial palsy, while other symptoms were reported less frequently. The most frequent neurological findings were limb paresis, sensory symptoms, and hypo/areflexia. Other frequent symptoms were facial palsy in about half, and bulbar palsy and respiratory dysfunction in about a quarter of cases. Frequencies of tetraparesis, sensory deficits, bulbar palsy and ataxia were higher in the probable/confirmed cases compared to overall proportions (**Table 4**). Separate data on tetraparesis vs paraparesis were reported in ten studies.[4, 16–18, 20, 21, 23, 25, 27, 31] Paraparesis was present in 69 of 251 reported cases (24% 95%CI 18–31). This included reports of cases with only lower limb weakness at nadir (30/251), cases with only lower limb weakness at an unclear time point in the disease (33/251), and cases that were reported as having a paraparetic variant of GBS (6/251). Heterogeneity in the analysis of all cases combined was substantial (I^2^ = 50–90%) for dysarthria, dysphagia, bulbar palsy, sensory deficits, areflexia/hyporeflexia, ataxia, respiratory dysfunction, and dysautonomia. In the probable/confirmed subgroup analysis this was substantial only for dysphagia and ataxia.

#### Diagnostic investigations

PCR, principally in serum, was the most frequently performed test for ZIKV diagnosis, although anti-ZIKV IgM was positive twice more often (**Table 5**). In the CSF, ZIKV PCR was positive in only 10 of 244 tested cases. Presence of neutralizing antibodies against ZIKV in the serum was tested in eight studies.[4, 15, 17, 23, 24, 26, 27, 31] To differentiate ZIKV from DENV, IgM antibodies against DENV were tested in 18 studies (426 cases), and were positive in 70 patients.[4, 14–18, 21, 23–33] Of these patients, 54 were also positive for ZIKV PCR, IgM and/or ZIKV neutralizing antibodies, and in 16 cases no separate information on ZIKV test results was available. Infection with CHIKV was investigated in nine studies and 187 cases, of which 16 were PCR or IgM positive.[14, 17, 19–21, 25, 28, 30]

**Table 5:** Ancillary investigations, treatment and disease progression of GBS cases associated with ZIKV reported in 21 case series.

Ancillary investigations	All cases (N 587)		Cases with probable/confirmed ZIKV infection (N 165)	
	**n/N**	**Pooled proportion (CI; I**^**2**^**)**	**n/N**	**Pooled proportion (CI; I**^**2**^**)**
**Zika virus certainty level**				
Confirmed	118/570	24% (11–40; 92%)	88/165	63% (32–90; 90%)
Probable	128/570	14% (3–30; 93%)	75/165	36% (9–67; 90%)
Suspected	324/570	44% (28–62; 92%)	-----	-----
**Arboviral tests**				
ZIKV infection^a^				
PCR (any sample)	118/470	30% (15–47; 90%)	88/153	71% (40–95; 88%)
PCR Serum	43/409	10% (1–24; 87%)	42/134	32% (5–66; 89%)
PCR CSF	10/244	3% (0–16; 74%)	6/78	11% (0–38; 80%)
PCR Urine	48/253	28% (7–54; 90%)	31/69	63% (21–97; 81%)
IgM (any sample)	254/375	68% (49–85; 90%)	126/137	97% (87–100; 52%)
IgM Serum	228/374	67% (45–85; 91%)	124/137	94% (81–100; 66%)
IgM CSF	36/111	60% (7–100; 95%)	33/50	77% (23–100; 91%)
PRNT ZIKV	121/154	86% (62–100; 86%)	23/23	100% (94–100; 0%)
PRNT ZIKV>DENV	20/105	16% (7–26; 14%)	11/18	67% (20–100; 52%)
DENV infection (PCR)	3/235	0% (0–1; 0%)	2/75	0% (0–10; 35%)
CHIKV infection (PCR or IgM)	16/187	1% (0–8; 56%)	4/88	0% (0–10; 29%)
DENV and CHIKV co-infection	6/165	1% (0–14; 71%)	2/84	0% (0–8; 42%)
**CSF analysis**	**425/537**	92% (79–100; 92%)	**122/139**	99% (87–100; 65%)
Increased protein level^b^	253/289	94% (89–98; 19%)	64/70	97% (89–100; 0%)
ACD	276/335	89% (80–96; 64%)	91/99	98% (92–100; 0%)
**Electrophysiological exam**	**245/477**	68% (49–85; 93%)	**86/145**	77% (46–98; 88%)
AIDP	143/244	62% (38–83; 89%)	62/86	68% (44–88; 59%)
AMAN	58/244	16% (0–41; 92%)	11/85	13% (1–33; 56%)
AMSAN	13/244	1% (0–6; 51%)	9/85	3% (0–11; 8%)
Equivocal	9/240	0% (0–2; 0%)	0/86	0% (0–0; 0%)
Unexcitable	4/240	0% (0–0; 0%)	1/86	0% (0–1; 0%)
Normal	11/245	0% (0–4; 26%)	2/86	0% (0–1; 0%)
**Immunomodulatory treatment**	**458/555**	92% (81–99; 88%)	**153/160**	100% (97–100; 8%)
IVIg	441/555	89% (77–97; 90%)	152/160	99% (94–100; 27%)
Plasma exchange	6/555	0% (0–0; 0%)	1/160	0% (0–0; 0%)
IVIg and plasma exchange	11/555	0% (0–1; 25%)	0/160	0% (0–0; 0%)
**Disease progression**				
Admission to ICU	287/544	49% (35–62; 86%)	82/146	57% (29–84; 86%)
Mechanical ventilation	118/567	21% (15–28; 44%)	35/140	19% (7–34; 57%)
Died	23/485	1% (0–3; 0%)	4/133	0% (0–2; 0%)

Abbreviations: ZIKV = Zika virus | CHIKV = Chikungunya virus | DENV = Dengue virus | PCR = polymerase chain reaction | CSF = cerebrospinal fluid | ACD = albuminocytological dissociation | AIDP = acute inflammatory demyelinating polyradiculoneuropathy | AMAN = acute motor axonal neuropathy | AMSAN = acute motor sensory axonal neuropathy | IVIg = intravenous immunoglobulin | ICU = Intensive Care Unit

^a^Proportions calculated per case, not per biological sample.

^b^Definition of increased protein level in CSF differed per study (>45 mg/dL, >51mg/dL or no cut-off reported).

Only five studies tested all ZIKV suspected cases for other infections that have been associated with GBS (*C*.*jejuni*, CMV, EBV, Hepatitis E virus, *Mycoplasma pneumoniae*).[4, 17, 23, 26, 31] And all tested cases (80/587; 14%) were negative for recent infection. None of the studies tested for all of these pathogens. Heterogeneity was considerable for all ZIKV laboratory tests (I^2^ = 75–100%).

**Fig 4:**
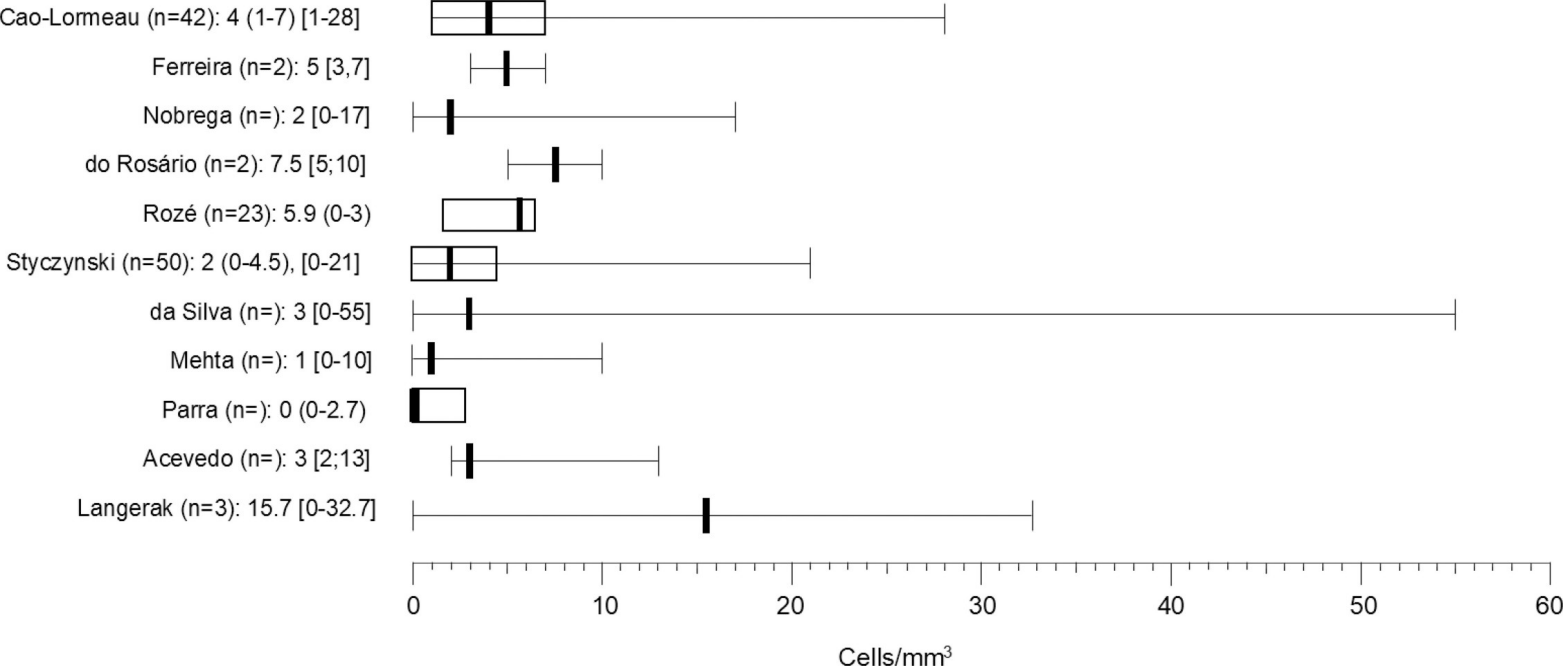
Overview of cell count in the CSF in reported studies. Cell count in medians, () = inter quartile range, [] = range.

CSF was examined in most studies, and information on protein level and cell count was provided by about half of these. Increased protein level and albuminocytological dissociation were present in the vast majority of cases and results were similar between all studies combined and the probable/confirmed subgroup. Eleven studies reported the CSF cell count, which did not exceed 55 cells/mm^3^, and medians were below 5 cells/mm^3^ (**Fig 4**).[4, 16–18, 20, 21, 26, 27, 29, 31, 32] Heterogeneity was limited for increased protein level and albuminocytological dissociation in all studies combined and the probable/confirmed subgroup.

Electrophysiological studies were done in about half of reported cases. In five studies no information on electrophysiological examination was reported.[16, 19, 28–30] Criteria used to classify cases into the different electrophysiological subtypes were reported in only five studies,[18, 23, 26, 31, 32] and included criteria by Hadden et al, Ho et al, and Rajabally et al.[50–52]. The most frequent electrophysiological subtype was AIDP in 62% (95%CI 38–83), followed by AMAN in 16% (95%CI 0–41), with both similar pooled proportions in the probable/confirmed ZIKV subgroup. In most studies, the majority of cases had an AIDP subtype, except for the study from French-Polynesia[4] where all cases were classified as AMAN, three studies with similar percentages of AMAN and AIDP,[20, 22, 27] a study from Singapore[15] with similar frequencies of AIDP and a normal EMG (in patients with MFS), and a Brazilian case series[21] reporting only a normal EMG and AMAN or AMSAN subtypes.

#### Treatment and disease progression

All but three studies[15, 20, 30] provided information on treatment, and in most studies almost all cases were treated with IVIg, except for three large studies, from Colombia[24, 32] and Brazil[19], where only 55–70% of patients were treated with immunomodulating therapy. Three studies provided no information on ICU admission,[23, 29, 30] which was necessary in about 50% of all reported cases, and even more frequent in the probable/confirmed subgroup (57%, 95%CI 29–84). Mechanical ventilation (MV) was necessary in about 20% of all cases and of the probable/confirmed subgroup. Death was infrequent in all cases combined and the probable/confirmed subgroup. Heterogeneity was substantial for immunomodulatory treatment and ICU admission, and moderate for MV (**Table 5**). We recalculated the pooled proportions of ICU, MV and death after excluding three studies that only selected cases admitted to the ICU,[17, 22, 28] and found that ICU admissions (261/518) were lower although still frequent (40%, 95%CI: 28–52), frequency of MV (111/441) was unchanged (22%, 95%CI: 16–28), and frequency of death (22/475) was similar (2%, 95%CI 0–4%), with comparable frequencies in the probable/confirmed subgroup analysis.

Eight studies informed about the time between onset and nadir of neurologic deficits (progressive phase), and only three studies reported the duration of the plateau phase (**Fig 3**). Only one large study from French-Polynesia informed about the functional evaluation of mobility of patients at nadir, showing incapacity to walk in 27/42 and difficulty to walk in 3/42.[4] The mobility of patients at 6 months after onset of disease was described in a study from Brazil[27] (33/50 walking without aid, 17/50 incapacity to walk) and a study from Puerto Rico[25] (48/79 able to walk 10 meters without aid, 39/79 any difficulty walking, and 12/79 incapacity to walk).

## Discussion

Our systematic review and meta-analysis show that published studies on ZIKV-related GBS typically report a classic sensorimotor type of GBS often with a facial palsy and a demyelinating electrophysiological subtype. The disease course is frequently severe with high rates of respiratory dysfunction and ICU admission. The time between onset of infectious and neurologic symptoms and negative PCR in most patients suggests a post-infectious rather than a direct infectious disease mechanism. These results should however be interpreted with caution as the studies included in this systematic review are variable in study design and setting, selection criteria, diagnostic ascertainment, and reporting of variables, which are potential sources of bias.

The combination of sensorimotor signs with facial palsy and respiratory insufficiency and a demyelinating electrophysiological subtype has previously been described in GBS patients with other preceding virus infections, such as CMV, indicating that such a clinical and electrophysiological profile may be related to preceding virus infections in general, in contrast to a bacterial infection with *C*.*jejuni*, that is associated with a pure motor axonal type of GBS.[7, 8, 53, 54] Additionally, although GBS is generally more common in men than in women, we found equal distributions of male and female frequencies in larger studies, similar to previous reports on GBS after other virus infections, suggesting that females may be more prone to virus-related GBS.[7, 53] This finding could however also be due to a higher incidence of ZIKV disease in females compared to males as has been shown in some studies.[55, 56] Another interesting finding was the high frequency of paraparesis (24%) compared to previous literature on GBS (1–11%), indicating that this may be a GBS variant related to ZIKV, although a lower percentage of paraparesis in the subgroup of patients with probable/confirmed ZIKV makes this feature less specific.[5, 57, 58] Furthermore, in some studies it is not clear if the paraparesis evolved to tetraparesis at a later time point, and whether myelitis, which has been linked to ZIKV in other studies, was excluded.[5, 57–59]

Some included studies diverged from the generally reported phenotype. Most importantly, the study from French Polynesia[4], in which all 42 patients had an AMAN electrophysiological subtype, 17 (40%) had a paraparesis and only 26 (62%) had hypo- or areflexia; and the study from Singapore[15], in which 4 out of 12 patients (33%) had MFS and one (8%) had MFS-GBS overlap syndrome. The high percentage of MFS in Singapore is in line with other publications that show high prevalence of MFS in Asian countries, but whether an AMAN subtype is typical for the Pacific region has not been studied.[5] As most of the other studies described cases from Latin America and the Caribbean, these discrepancies may be due to regional differences in host and/or environmental factors, including differences in the ZIKV strains.[5, 60] However, some dissimilarities could also be due to differences in diagnostic and electrophysiological accuracy between studies. For instance, the interpretation of electrophysiological data in the study from French Polynesia[4] has previously been questioned, as the prolonged distal motor latencies, found at first examination and persisting after 4 months, would be more consistent with the AIDP subtype.[61]

The median time between the onset of infectious symptoms and the start of neurologic symptoms varied between 5 and 12 days, which is similar to other infections preceding GBS.[7, 62, 63] Considering that the incubation period of ZIKV infection is estimated at 1–2 weeks, the latency between ZIKV infection and GBS was more than a week for most cases, suggesting a post-infectious immunopathogenesis, rather than direct neuronal damage or a para-infectious mechanism, as has been suggested in previous publications.[64, 65] A low frequency of ZIKV PCR positivity in blood and CSF, and a low cell count in the CSF in the majority of cases, further argues against a direct infection. These findings are in line with an *in vivo* study that showed resistance of peripheral nerve cells to infection by ZIKV.[66]

Remarkably, half of all cases combined and more than a half of probable/confirmed cases were admitted to the ICU. This proportion is higher than expected based on other literature (15–30%)[67, 68], and remained higher (40%) after we excluded papers that only included patients admitted to the ICU. These data may indicate that GBS following ZIKV infection is often severe enough to necessitate ICU admission. However, the percentage of mechanically ventilated patients (20%) is similar to most other publications.[5, 58, 69, 70] It is not clear what causes this discrepancy. A possible explanation is that presence of autonomic symptoms, rapid progression, severe weakness, or respiratory problems that did not evolve into respiratory insufficiency, were reasons to admit to the ICU, especially during the ZIKV epidemic when an increased vigilance for GBS may have lowered the threshold for intensive care monitoring. Furthermore, many studies were done in specialized centres that may receive more severely affected patients referred from other centres, or may more easily admit patients to the ICU for monitoring compared to non-specialized centres.

The large variability of study designs and settings, selection criteria, diagnostic ascertainment and citation of variables were important sources of bias within studies and heterogeneity across studies, which is a critical limitation of our meta-analysis. Most importantly, diagnostic ascertainment of GBS and ZIKV differed, and electrophysiological criteria were not reported in most studies. Diagnostic certainty of ZIKV infection was limited in most studies, and other preceding infections in GBS were often not excluded. Furthermore, the type of hospital may have biased the inclusion of severe cases, causing heterogeneity in both clinical signs and disease progression. We calculated the I^2^ to quantify this heterogeneity between studies, and have performed a sensitivity analysis to estimate the pooled frequencies among a subgroup of cases with only probable/confirmed ZIKV to analyse the clinical picture of GBS among cases with a higher ascertainment of ZIK infection.

The I^2^ was considerable for most infectious symptoms, which is likely due to recall and reporting bias, and as we assumed infectious symptoms were absent, rather than missing, if not reported, we may have increased this heterogeneity. Heterogeneity in neurologic symptoms and signs was considerable for some variables, which may be due to differences in study design and methodology and geographical location. Heterogeneity of arboviral test results was also considerable, which may be due to differences between timing of sample collection and variation in incubation and viremia periods. In general, the variables with considerable heterogeneity are difficult to interpret and preclude any firm conclusions to be drawn from these data. However, the I^2^ in the probable/confirmed ZIKV subgroup was generally lower than in all cases combined, indicating that the heterogeneity was partly caused by differences in the diagnostic certainty of ZIKV infection, providing more evidence for a specific clinical and electrophysiological phenotype of ZIKV-related GBS.

## Conclusion

Published studies on ZIKV-related GBS generally report a sensorimotor demyelinating GBS with a frequent facial palsy and a severe disease course that often necessitates ICU admittance. The paraparetic variant of GBS is also common, which should caution clinicians to exclude myelitis in ZIKV-related cases. The time between onset of infectious and neurologic symptoms and absence of viral genome detected by PCR in most cases suggest a post-infectious, rather than a direct infectious or para-infectious mechanism.

## Supporting information

S1 Text PRISMA checklist. Preferred Reporting Items for Systematic Reviews and Meta-Analyses (PRISMA) checklist.
(DOC)

S2 Text Protocol data extraction. Protocol used for data extraction of the selected papers.
(DOCX)

S1 Fig PRISMA flowchart. Preferred Reporting Items for Systematic Reviews and Meta-Analyses (PRISMA) flowchart (idem to Fig 1).
(TIF)

S2 Fig Overall pooled proportions (forest plots) of ZIKV infection certainty levels in reported GBS cases. (TIF)

S1 Data Data extraction sheet. Excel sheet showing the data as extracted from the selected papers. All cases combined and the cases with probable or confirmed Zika virus infection as displayed separately.
(XLSX)
