# Supplementary material for: Guillain-Barré syndrome related to Zika virus infection: A systematic review and meta-analysis of the clinical and electrophysiological phenotype
Source: PLoS Negl Trop Dis. 2020 Apr 27;14(4):e0008264. doi: 10.1371/journal.pntd.0008264 (PMC7205322; doi:10.1371/journal.pntd.0008264)
Supplement: S2 Text — Protocol used for data extraction of the selected papers. (DOCX) [file pntd.0008264.s002.docx]

**Dictionary and Directions for Extracting Data of the Systematic review on clinical characteristics and prognosis of Guillain-Barré Syndrome after Zika viruses** *(PROSPERO 2018 CRD42018081959)*

**STUDY IDENTIFICATION**

- **SETTING**: describe if GBS cases were selected in a single healthcare service (single centre) or several (multicentre), in the community or from a surveillance reporting.

**DEMOGRAPHIC DATA**

For continuous values, we register the median (IQR: min – max) or the average (IC: min – max); but in case series, without estimates of median/average, we report the central value with the range of values [min – max].

**ACUTE SYMPTOMS OF THE PRIOR INFECTIOUS DISEASE**

In the numerator, we report the number of cases with the specific symptom, and in the denominator, we report the number of cases reported OR, if it is not informed, the total number of cases.

We are attributing the zero value (0) when there is no report of the symptom or when the authors inform the absence of the symptom.

**LABORATORY INVESTIGATIONS**

In the numerator, we report the number of cases with positivity for the specific test, and in the denominator, we report the number of patients who were tested.

We are attributing the symbol * when there is no information about the test.

- **PRNT_ZIKV>DENV**: titres of antibody neutralization test for ZIKV greater than for DENV (describe the titre ratio when this information is available).
- **PRNT_ZIK≤DENV**: titres of antibody neutralization test for ZIKV less than or equal to DENV (describe the titre ratio when this information is available).
- **Neg_OthersVirBac**: we register when there is a stating of culture, PCR, antigen or IgM test negative for the specific microorganism, putting the number of cases with negativity for the specific test in the numerator, and the number of patients who were undergo to the test in the denominator.

**DIAGNOSIS OF ZIKV IN GBS CASES**

- **ZIKV_CONFIRMED**:
  - detection of ZIKV PCR, culture, viral antigen in serum, CSF, tissue or other specimen (saliva, urine, semen), **OR**
  - ZIKV IgM positive in serum or CSF **AND** PRNT positive for ZIKV and negative for DENV (or other flavivirus epidemic at moment and time of study).
- **ZIKV_PROBABLE**:
  - ZIKV IgM positive in serum or CSF, **AND**
  - PRNT positive for ZIKV and DENV (or other flavivirus epidemic at moment and time of study) or DENV IgM negative with no PRNT performed.
- **ZIKV_SUSPECTED**: symptoms of ZIKV infection* or associated disease (stated by the authors or according to symptoms described) within 3 months before neurologic disease, with or without serologic evidence of ZIKV (not meeting confirmed or probable criteria), for example IgG or IgM positivity OR GBS patient reported during ongoing epidemic in the region reported.
- **See CDC Zika virus case definition:** <https://wwwn.cdc.gov/nndss/conditions/zika/case-definition/2016/06/>
- **CHIKV COINFECTION**: detection of CHIKV infection by IgM or PCR in serum, CSF, tissue or other specimen (saliva, urine, semen).
- **DENV COINFECTION:** detection of DENV by PCR, culture, antigen in serum, CSF, tissue or other specimen (saliva, urine, semen).
- **FLAVIVIRUS_RECENT_INSPEC:** any laboratory evidence of recent flavivirus infection, defined as IgM or PRNT positivity for ZIKV, DENV, that does not fall under category ZIKV_CONFIRMED, ZIKV_PROBABLE, CHIKV COINFECTION OR DENV COINFECTION (this category has not been used in the final manuscript)

**RESULTS CSF ANALYSIS**

In the numerator, we report the number of cases with positivity for the specific test, and in the denominator, we report the number of patients who were undergo test.

For continuous values, we register the median (IQR: min – max) or the average (IC: min – max), but in case series, without estimates of median/average, we can report all values in a rising order or the central value with the variation [min – max].

- **ALBUMINOCYTOLOGIC DISSOCIATION**: measurements of protein > 52 mg/dL AND cells < 50 cell/mm^3^ in a sample.

**NEUROLOGICAL SYMPTOMS**

In the numerator, we report the number of cases with the specific symptom at admission or during the hospital stay, and in the denominator, we report the number of patients in which the symptom/sign is reported OR, if it is not informed, the total number of cases.

For continuous values, we register the median (IQR: min – max) or the average (IC: min – max), but in case series, without estimates of median/average, we can report the central value with the range of values [min – max].

We are attributing the zero value (0) when the authors inform the absence of the symptom. If the report of the symptoms is detailed (as usually displayed in case reports), but a certain variable (e.g. sensory signs) is not mentioned, we will presume it is absent and attribute the zero (0) value. If the report is not detailed and variables are not mentioned, we will attribute the symbol *.

We also are attributing the symbol * when there is no information about a kind of category of a reported symptom, as an example, when there is reporting of weakness but no information if it was ascending, we must fill ASCENDING WEAKNESS with ‘*’

- **mDiasInicioSimtZIKV_NEURO**: means the median (IQR: min – max) or average (IC: min – max) of days between the start of symptoms of ZIKV disease and neurological symptoms.
- **nDiasInicioSimtZIKV_NEURO**: number of cases with information on days between the start of symptoms of ZIKV disease and neurological symptoms.
- **DYSARTRIA:** speech difficulty informed as a symptom, but not reported as a finding in the neurological examination.
- **SWALLOWING DIFFICULTY:** informed as a symptom, but not reported as a finding in the neurological examination.
- **DIPLOPIA**: double vision informed as a symptom, but not reported as a finding in the neurological examination.
- **DIFFICULTY TO WALK**: means gait difficult or difficulty walking without aid, but no not-walking, which must to be register as UNABLE TO WALK.
- **UNABLE TO WALK**: means inability to walk without aid.

**NEUROLOGICAL EXAMINATION**

Findings at any time during the disease are registered.

In the numerator, we report the number of cases with the specific symptom, and in the denominator, we report the number of patients with this information.

For continuous values, we register the median (IQR: min – max) or the average (IC: min – max), but in case series, without estimates of median/average, we can report all values in a rising order or the central value with the variation [min – max].

We are attributing the zero value (0) when there is no report of the symptom or when the authors inform the absence of the symptom.

We are attributing the symbol * when there is no report about a score or about a classification of a reported symptom.

If the report of the symptoms is detailed (as usually displayed in case reports), but a certain variable (e.g. ataxia) is not mentioned, we will presume it is absent and attribute the zero (0) value. If the report is not detailed and variables are not mentioned, we will attribute the symbol *.

- **NERVO CRANEANO BULBAR:** speech and/or swallowing difficulties as detected in the neurological examination, but no just speech/swallowing difficulty informed as symptoms, which must be registered in NEUROLOGICAL SYMPTOMS, as DISARTRIA OR SWALLOWING DIFFICULTY, respectively.
- **NERVOS CRANEANOS III OR IV OR VI**: abnormality of oculomotor function as detected in the neurological examination, but not just the information of diplopia, which must be registered in NEUROLOGICAL SYMPTOMS, as DIPLOPIA
- **TETRAPARESIS**: means weakness detected in the 4 limbs.
- **PARAPARESIS_MMII**: means weakness detected in the lower limbs, but not in the upper limbs
- **OnlyPARESIS_MMSS**: means weakness detected in the upper limbs, but no weakness in the lower limbs
- **mDiasInicioSimptNEURO_Nadir**: means the median (IQR: min – max) or average (IC: min – max) of days between the start of neurological symptoms and the worst neurological deficits.
- **mDiasPLATÔ_NEURO**: means the median (IQR: min – max) or average (IC: min – max) of days between the worst neurological deficit and the start of improvement of disease.

**DIAGNOSTIC CRITERIA**

In the numerator, we report the number of cases with the specific Brighton level, and in the denominator, we report the number of patients with the information on Brighton Criteria. We are attributing the symbol * when there is no information about it.

- **BRIGHTON LEVEL 1-2**: number of cases with Brighton level 1 or 2.
- **BRIGHTON LEVEL 1-3**: number of cases with Brighton level 1, 2 or 3.
- **BRIGHTON LEVEL 1**: clinical and EMG criteria plus albumin-cytologic dissociation in CSF (protein > 52 mg/dL AND cells < 50 cell/mm^3^ in a sample).
- **BRIGHTON LEVEL 2**: clinical criteria plus cell< 50/mm^3^ in CSF OR EMG criteria.
- **BRIGHTON LEVEL 3**: only clinical criteria without other explanation for the clinical picture= flaccid weakness with absence or reduction of reflexes in limbs and a monophasic pattern within 12 hours to 28 days of the nadir.
- **OUT FROM BRIGHTON CRITERIA (BRIGHTON LEVEL 4)**: suspicion of GBS without other explanation for the clinical picture but no clinical criteria for fulfil the Level 3 of Brighton, for instance, presence of hyperreflexia
- **Miller Fisher Syndrome:** can be classified according to Brighton criteria, but should also be noted if present in this separate variable
- **Other variants of GBS:** pure motor, paraparetic, pharyngo-cervico-brachial, pure sensory, Bickerstaff Brainstem Encephalopathy, Miller Fisher-overlap syndrome

**OUTCOME**

In the numerator, we report the number of cases with the specific outcome, and in the denominator, we report the number of patients with this information.

For continuous values, we register the median (IQR: min – max) or the average (IC: min – max), but in case series, without estimates of median/average, we can report all values in a rising order or the central value with the variation [min – max].

We are attributing the symbol * when there is no information about the specific symptom.

The **GBS or Hughs Disability Scale** categorizes patients regarding their ability to walk: **0**- healthy; **1**- minor symptoms but able to run; **2**- able to walk 10m without aid; **3**- able to walk 10m with aid; **4**- bedridden/chairbound; **5**- assisted ventilation; **6**- dead.
