# Supplementary figures and images for: Guillain-Barré syndrome related to Zika virus infection: A systematic review and meta-analysis of the clinical and electrophysiological phenotype
Source: PLoS Negl Trop Dis. 2020 Apr 27;14(4):e0008264. doi: 10.1371/journal.pntd.0008264 (PMC7205322; doi:10.1371/journal.pntd.0008264)

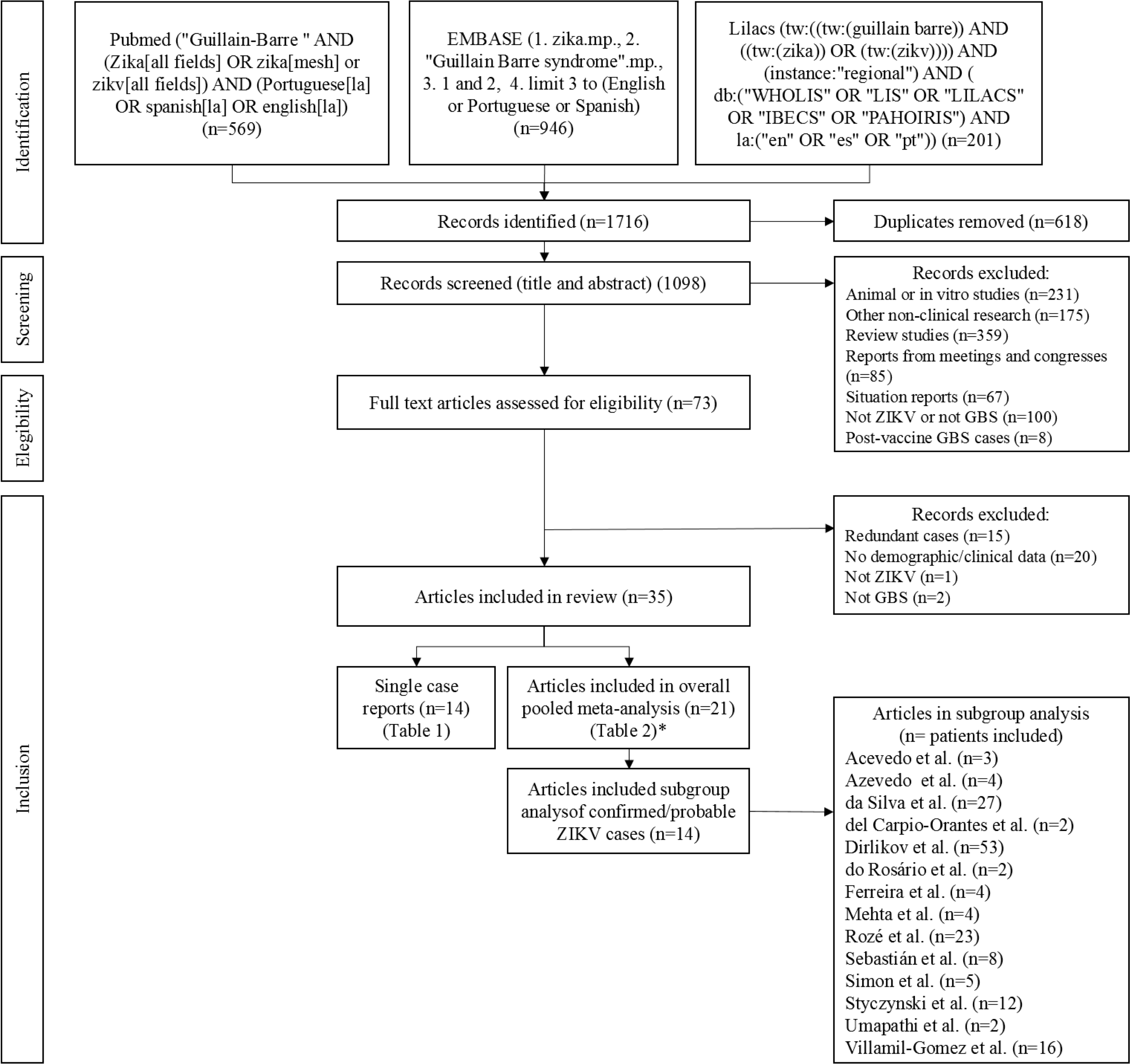

Supplement: S1 Fig — Preferred Reporting Items for Systematic Reviews and Meta-Analyses (PRISMA) flowchart (idem to Fig 1). (TIF) [file pntd.0008264.s003.tif]

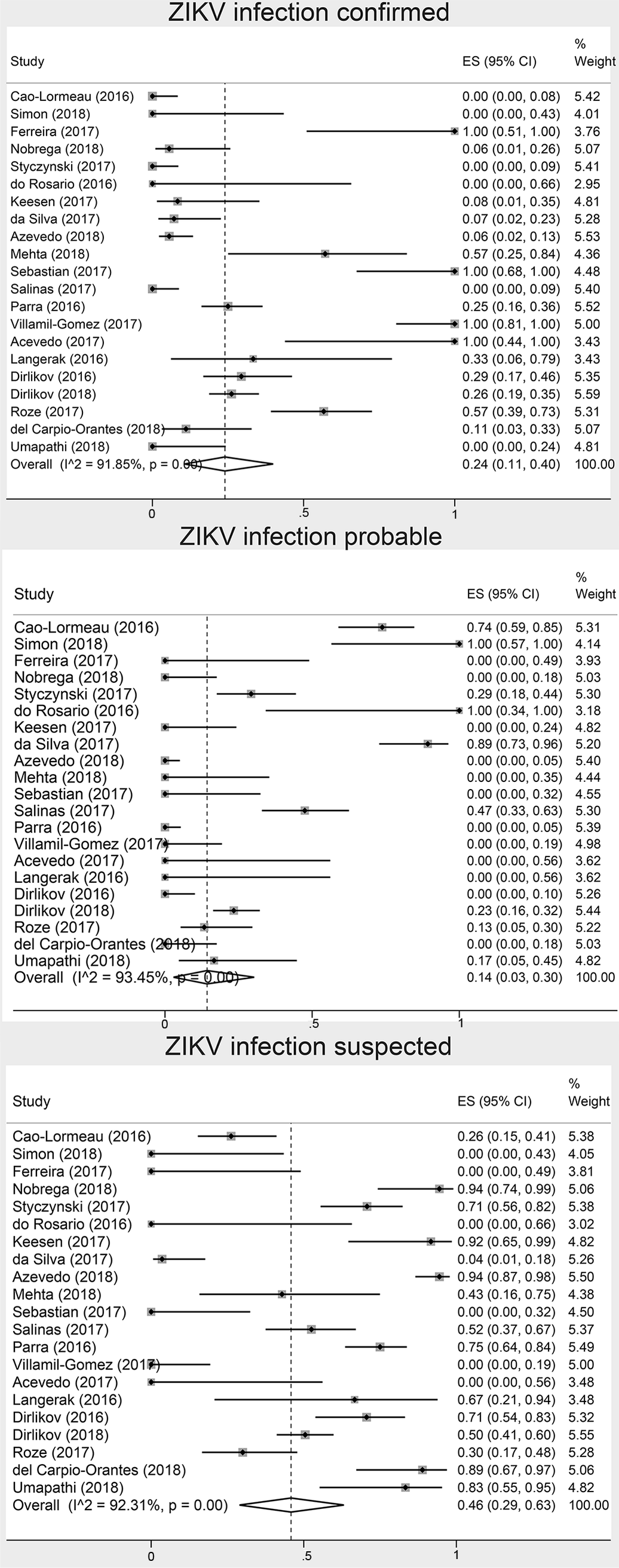

Supplement: S2 Fig — (TIF) [file pntd.0008264.s004.tif]
